# Grouping miRNAs of similar functions via weighted information content of gene ontology

**DOI:** 10.1186/s12859-016-1367-0

**Published:** 2016-12-22

**Authors:** Chaowang Lan, Qingfeng Chen, Jinyan Li

**Affiliations:** 1 School of Computer, Electronic and Information, and State Key Laboratory for Conservation and Utilization of Subtropical Agro-bioresources, Guangxi University, No.100 Daxue Road, Nanning, 530004 China; 2 Advanced Analytics Institute, Faculty of Engineering and IT, University of Technology Sydney, PO Box 123, Broadway, Sydney, NSW 2007 Australia

**Keywords:** Gene ontology, Functions of miRNAs, Information content, GO graphs, Spectral clustering

## Abstract

**Background:**

Regulation mechanisms between miRNAs and genes are complicated. To accomplish a biological function, a miRNA may regulate multiple target genes, and similarly a target gene may be regulated by multiple miRNAs. Wet-lab knowledge of co-regulating miRNAs is limited. This work introduces a computational method to group miRNAs of similar functions to identify co-regulating miRNAsfrom a similarity matrix of miRNAs.

**Results:**

We define a novel information content of gene ontology (GO) to measure similarity between two sets of GO graphs corresponding to the two sets of target genes of two miRNAs. This between-graph similarity is then transferred as a functional similarity between the two miRNAs. Our definition of the information content is based on the size of a GO term’s descendants, but adjusted by a weight derived from its depth level and the GO relationships at its path to the root node or to the most informative common ancestor (MICA). Further, a self-tuning technique and the eigenvalues of the normalized Laplacian matrix are applied to determine the optimal parameters for the spectral clustering of the similarity matrix of the miRNAs.

**Conclusions:**

Experimental results demonstrate that our method has better clustering performance than the existing edge-based, node-based or hybrid methods. Our method has also demonstrated a novel usefulness for the function annotation of new miRNAs, as reported in the detailed case studies.

## Background

MiRNA is a small non-coding RNA molecule highly conserved in plants and animals. Many investigations have reported that miRNAs can play important roles in various vital biological processes such as gene expression, cell development, cancer progression, and immune process by binding to the 3^′^ untranslated regions of their target genes, which can result in the translational repression or rapid degradation of the target transcripts [1]. As miRNA function is usually carried out by groups of miRNAs rather than individually [2], clustering miRNAs for the function annotation of new miRNAs is a problem of wide interests, given that the knowledge of co-regulating miRNAs is limited in wet-labs.

Sequence or structure-based similarity measurements have been previously proposed to cluster miRNAs for similar functions. For example, the Rfam [3] and miRBase [4] databases use sequence similarities to classify the functions of miRNAs. The concern is that some miRNAs having a high sequence similarity may have distinct functions. Also, the structure-function relationships used in the function annotation of miRNAs have been reported to show serious limitations in the case of complex substructures [5].

Recently, individual target genes of differentially expressed miRNAs have been explored for clustering miRNAs into groups of similar functions. However, a miRNA can regulate multiple target genes. To overcome this limitation, we explore a novel similarity measurement between the two sets of target genes corresponding to two miRNAs. We propose to transfer the function similarity between the two sets of target genes as the function similarity of the two miRNAs.

The function similarity between two sets of target genes has been previously investigated and can be derived from the structure information of gene ontology (GO) trees of these target genes [6]. The hierarchical structure of a GO tree is a directed acyclic graph (DAC), containing structured vocabularies to describe the functions at different levels of the gene products [7]. The nodes of a GO tree are called *terms*. An *edge* in a GO tree represents a relationship between two terms. The two most common relationships between two terms are *is_a* for subclass and *part_of* for component [8]. GO terms, their relationships and the similarity between two GO trees have been considered in many bioinformatics applications by literature such as for pathway analysis [9], gene network analysis [10], and gene expression research [11].

We introduce a novel measurement of information content, a weighted information content of gene ontology, to estimate the similarity between two GO trees. The weighted information content of a term in a GO tree is determined by three factors: the number of descendants of the term, the depth of the path from the term to the root node or to the most informative common ancestor (MICA), and the relationships along the edges in the path. Every term in a GO tree has its unique information content. Based on this definition of information content, the similarity between two GO trees is proposed to be measured by the information contents of all the common terms between the two GO trees, relative to the information contents of all the unique terms. Two GO trees are more similar in function than others if they have more common terms and fewer unique terms. When we are given two sets of GO trees, the similarity between the two sets are derived by computing all the pair-wise similarities of the GO trees from the two sets. This similarity between the two sets of GO trees is then transferred as a similarity measurement between the two miRNAs whose target genes correspond to the two set of GO trees.

In the literature, node-based [12] and edge-based methods [13] have been proposed to measure the similarity between GO trees or subtrees. By their definitions, the nodes in the same hierarchy are assumed to have an equal distance to the root, an idea which was criticized by [14]. Further, the information content of a term in a GO graph is exactly the same as another’s, even if the two terms have different depths in the graph [15]—it ignores important properties of edges such as the depth and the topology information of the term in the GO graph. Node-based methods also focus on the most informative common ancestor like our method, but they neglect the whole path structures of GO terms. Moreover, the edge-based methods do not distinguish the weight of terms at different depths of a GO graph. Our weighted information content of gene ontology can overcome these shortcomings.

For enhancing the performance on clustering the miRNAs into subgroups of similar functions, a self-tuning technique is applied to determine the optimal parameter *σ* for the spectral clustering method [16]. Further, an appropriate cluster number is estimated by the eigenvalues of the normalized Laplacian matrix. Our approach has been used for grouping miRNAs of similar functions associated with diseases stored at several databases. Most of the experimental results showed good accuracy and the annotation results for new miRNAs can be supported by evidence found from the other databases or from recently published literature.

## Methods

MiRNAs and their target genes were downloaded from http://mirtarbase.mbc.nctu.edu.tw/php/download.php (the file hsa_MTI.xls). This file is a relational table having 39110 lines and 9 columns: miRTarBase ID, miRNA, Species (miRNA), Target Gene, Target Gene (Entrez ID), Species (Target Gene), Experiments, Support Type, and References (PMID). Each line of this table stores information of one miRNA and the information of one of its target genes. We note that some multiple lines in this table actually refer to the same miRNA—researchers have done many different experiments to confirm the same miRNA’s target genes. As a result, there are only 289 distinct miRNAs in this file. We used all of them in this work. The disease information associated with each of the miRNAs was searched at the HMDD database (http://www.cuilab.cn/hmdd). Out of the 289 miRNAs, 24 did not have disease information available.

The GO terms of a target gene were searched at the EMBL-EBI website (http://www.ebi.ac.uk/). The relationships (i.e., is_a and part_of) of these GO terms were derived from the AmiGo database (http://amigo.geneontology.org/amigo). These GO terms and their relationships were integrated and represented by graphs.

### **Definition 1.**

**GO graph of a gene**. Given a gene *g*, its GO terms and the relationships of these GO terms are represented by a DAC (direct acyclic) graph *G*(*g*)=(*T* *e* *r* *m* ^*g*^,*E* *d* *g* *e* ^*g*^), where *T* *e* *r* *m* ^*g*^ represents the set of nodes each labeled with a GO term, and *E* *d* *g* *e* ^*g*^ represents the set of edges each labeled with a relationship (is_a or part_of) between a pair of terms of *g*. Such a graph is also called a GO graph or GO tree of *g*.

### **Definition 2.**

**Root node**. The root node of a GO graph is the term node which has an in-degree only. A GO graph has one and only one root node.

### **Definition 3.**

**Leaf nodes**. A leaf node of a GO graph is a term node which has an out-degree only. The GO graph of a gene may have multiple leaf nodes.

**Fig. 1:**
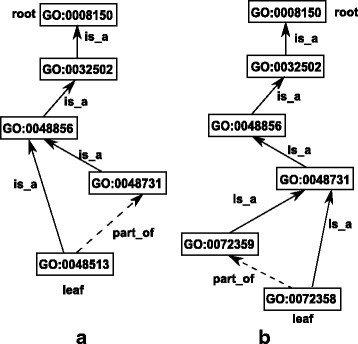
**a** The GO graph of GO:0048513 and **b** The GO graph of GO:0072358. Each includes one leaf term only

**Fig. 2:**
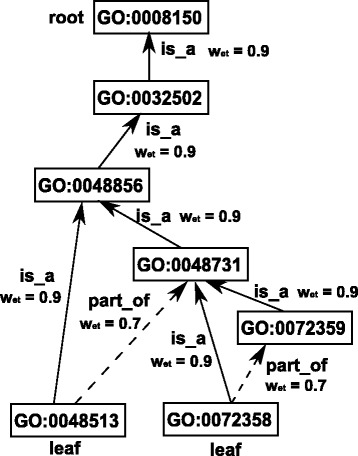
A GO tree integrating graphs of GO:0048513 and GO:0072358

Figure 1 a, b and Fig. 2 show three examples of GO graphs, where root nodes, leaf nodes, and the relationships of some pairs of terms are explained.

### **Definition 4.**

**Term graph**. A term graph is a special form of GO graph. Given a GO graph, if it has only one leaf term *A*, such a GO graph is called *A*’s term graph, denoted by *T* *G*_*A*_=(*A*,*T* *e* *r* *m*_*A*_,*E* *d* *g* *e*_*A*_), where *T* *e* *r* *m*_*A*_ and *E* *d* *g* *e*_*A*_ represent the set of GO terms and the set of edges of the GO graph, respectively.

Given a GO graph *G*=(*T* *e* *r* *m*,*E* *d* *g* *e*), for every term *t*∈*T* *e* *r* *m*, we can construct one term graph *T* *G*_(*t*,*G*)_=(*t*,*T* *e* *r* *m*_(*t*,*G*)_,*E* *d* *g* *e*_(*t*,*G*)_), where *T* *e* *r* *m*_(*t*,*G*)_ is the set of terms in the path from *t* to the root node of *G*, and *E* *d* *g* *e*_(*t*,*G*)_) is the set of edges in the path from *t* to the root node of *G*.

In particular, a leaf node *l*_*n* *o* *d* *e* of GO graph *G* can form a leaf term graph ${TG}_{(l\_node, G)} =(l\_node$, ${Term}_{(l\_node, G)}, {Edge}_{(l\_node, G)})$. Leaf term graphs of a GO graph are used later to define the similarity between two GO graphs. The subscript *G* is sometimes omitted when it is understood. Figure 1 a and b are actually the two leaf term graphs of Fig. 2.

### **Definition 5.**

**Depth and level of a node**. The depth of a term node *t* in a GO graph is the number of edges in the longest path from *t* to the root node of the graph. For example, the depth of 0048513 is 4 as shown in Fig. 1 a. If the depth of a term is *d*, the term is also said to be at level *d*.

Given two term graphs *T* *G*_*A*_=(*A*,*T* *e* *r* *m*_*A*_,*E* *d* *g* *e*_*A*_) and *T* *G*_*B*_=(*B*,*T* *e* *r* *m*_*B*_,*E* *d* *g* *e*_*B*_). There may exist many common terms (at least the root node) between *T* *e* *r* *m*_*A*_ and *T* *e* *r* *m*_*B*_. For example, term 0048856 is a common term between *T* *G*_0048513_ (Fig. 1 a) and *T* *G*_0072358_ (Fig. 1 b). For all other terms in *T* *e* *r* *m*_*A*_ or *T* *e* *r* *m*_*B*_, they are called *uncommon* or unique terms.

## Clustering miRNAs for similar functions

Suppose we are given *h* number of miRNAs, the first process of our method is to construct a *h*×*h* similarity matrix of these miRNAs. For every pair of miRNAs in the matrix, their similarity is transferred from the similarity between their two sets of target genes. As every gene can be represented by a GO tree, the similarity between the two sets of target genes can be determined by computing the similarity between the two sets of GO trees. With this *h*×*h* similarity matrix as input, we use a spectral clustering method to group miRNAs of similar functions. We present details for these steps:

1. Compute the weighted information content of every term in a GO graph to determine the similarity between two GO trees;
2. Compute the similarity between two sets of GO trees to determine the similarity between two miRNAs;
3. Construct a similarity matrix of the *h* miRNAs, and subgroup them for a similar function in each group using the similarity matrix as input.

**Fig. 3:**
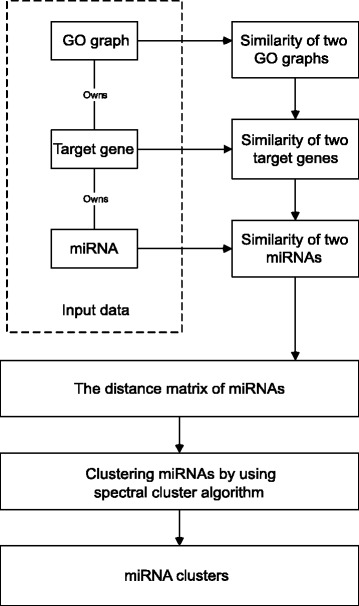
Our method for clustering miRNAs. The raw input to the method consists of data of miRNAs, the target genes of the miRNAs, and the GO graphs of the miRNAs’ target genes. Firstly, the similarity of two GO graphs is computed according to the weighted information content. Secondly, the similarity of two target genes of two miRNAs is calculated by using the similarity of their GO graphs. Thirdly, the similarity between two miRNAs is computed by their similarity of their target genes. Then the miRNA distance/similarity matrix is constructed by these similarity scores. Finally, the spectral clustering method is applied to cluster the miRNAs

The framework of our method is showed in Fig. 3.

### Compute a weighted information content of a term in a GO graph

The traditionally defined information contents of two terms in a GO graph can be exactly the same even if the two terms have different depths in the graph [17]. We propose a new measurement for the information content to deal with this issue. It is a descendant-based information content, adjusted by a weight proportional to the depth and the relationships of the nodes in the path of the term to the root node.

For a GO graph *G*=(*T* *e* *r* *m*,*E* *d* *g* *e*), the information content (*IC*) of a term *t*∈*T* *e* *r* *m* is computed by

1 $$ IC(t) = -\log\frac{1+\| descends(t) \|}{\| Term \|}  $$

where ∥*d* *e* *s* *c* *e* *n* *d* *s*(*t*)∥ is the number of *t*’s descendants in *G*, and ∥*T* *e* *r* *m*∥ is the number of terms of *G*. This equation implies that a parent node’s *IC* is always smaller than its child node (i.e., a GO term closer to the root node has a smaller *IC* value); and that two different leaf terms have the same *IC* (because they do not have any descendants). For example, term 0048731 in Fig. 2 has 3 descendants (0072359, 0072358, and 0048513). Its *IC* is $-\log (\frac {1+3}{7})= 0.243$. The *IC* values of the other terms in Fig. 2 are listed in Table 1.

**Table 1:** The *IC*, length weights, edge weight, and weighted information content of the terms in Fig. 2

Term	GO:0008150	GO:0032502	GO:0048856	GO:0048731	GO:0072359	GO:0048513	GO:0072358								
*IC*	0	0.067	0.146	0.243	0.544	0.845	0.845								
*ω*_*depth*_(*t*,*G*)	1	0.9	0.81	0.729	0.9	0.9	0.63								
*ω*_*edge*_(*t*,*G*)	0	0.533	0.567	0.599	0.533	0.533	0.642								
*ω* *I* *C*(*t*,*G*)	0	0.189	0.288	0.382	0.538	0.671	0.737								

#### **Definition 6.**

**The most informative common ancestor.** Given a term graph, if a leaf term can reach to a node by walking through a direct line, this node is called an ancestor term of the leaf term. A common ancestor term is such an ancestor term that two input leave terms can both reach. The most informative common ancestor (MICA) is the common ancestor term that has the maximum *IC* value of two term graphs.

These information contents are then adjusted by a weight of the path of the term to the root node or to the MICA node. The weight is named *edge weight* which is determined by two factors: the relationships of the edges in the path and the distance of the path. Let *T* *G*_*A*_={*A*,*T* *e* *r* *m*_*A*_,*E* *d* *g* *e*_*A*_} and *T* *G*_*B*_={*B*,*T* *e* *r* *m*_*B*_,*E* *d* *g* *e*_*B*_} be two term graphs, *G* be the merged graph, and *mica* be the two term graphs’ MICA. For a term *t* in the graph *G*, its distance weight *ω*_*edge*_(*t*,*G*) is defined as

2 $$ \omega_{edge}(t, G) = \frac{2}{\pi}* \arctan\frac{1}{\omega_{depth}(t, G)}  $$

where *ω*_*depth*_(*t*,*G*) is 1 if *t* is the root node. If *t* is *mica*’s ancestor or *mica*, *ω*_*depth*_(*t*,*G*) is the product of all the relationships in the longest path from *t* to the root node of *T* *G*_*A*_ or *T* *G*_*B*_, otherwise it is the product of all the relationships in the longest path from *t* to the *mica*.

The *is_a* relationship is more important than the *part_of* relationship. Thus, we set *is_a* as W _*edge*_= 0.9 and *part_of* is set as W _*edge*_= 0.7. We note that the edge weight of a term increases when the term is farther to the root node or to the MICA. The arctan transformation is to standardize the reciprocal of two length weights as they can be very large.

For example, *ω*_*depth*_ of term 0008150 in Fig. 2 is 1, since it is the root node of the GO tree. The *ω*_*depth*_ value of its child 0032502 is 0.9, as the relationship between these two terms is *is_a*. The *ω*_*depth*_ value of *G* *O*:0072359 is 0.9, because this term is MICA’s descendant term and the relationship between MICA and this term is *is_a*. The other terms’ *ω*_*depth*_ values are listed in Table 1. We note that if a term has multiple longest paths to the root node or MICA, we choose the one which provides the biggest edge weight for the term. The edge weights of the terms in Fig. 2 are also listed in Table 1 (see the second-last row).

By Eq. 2, if an ancestor term of the MICA is near to the root, this term contributes less similarity to the two term’s trees as it is more general. For a descendant term of the MICA, which is near to MICA, contributes less dissimilarity. Unlike traditional edge-based methods [18] which set all the edges as the same weight, our method considers both the distance of the terms to the root or MICA node and the difference between *is_a* and *part_of* to measure the distance weight of a term.

We combine the initial information content (i.e., Eqn. 1) of a term *t* in a merged GO graph *G* and its edge weight (i.e., Eqn. 2) to derive a weighted information content for the term. It is denoted by *ω* *I* *C*(*t*,*G*), defined as

3 $$ \omega IC(t, G) = \sqrt{IC(t) \ast \omega_{edge}(t, G)}  $$

The weighted information contents of all the terms in Fig. 2 are shown in the last row of Table 1.

By this definition, only the root node has a weighted information content of 0. It is understandable because a root node does not contribute to the weight—it has no parent node and it is the ancestor of all other terms. As some terms (e.g., the leaf nodes of a graph) having the same *IC* can occur at different levels of the graph, the *IC* value alone cannot reflect the different importance of these terms. This is the main reason why edge weights are used to resolve this issue.

### Determine the similarity of two genes based on weighted information content

As the GO graph of a gene may contain multiple leaf term graphs, we first define the similarity between two term graphs, and then define the similarity between two GO graphs.

**Fig. 4:**
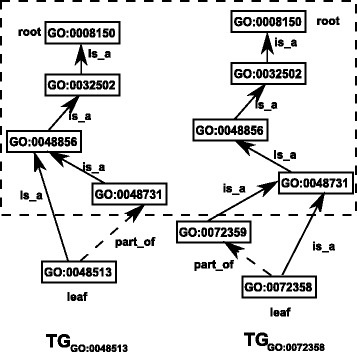
A schematic illustration of the similarity between two graphs

Given two term graphs *T* *G*_*A*_=(*A*,*T* *e* *r* *m*_*A*_,*E* *d* *g* *e*_*A*_) and *T* *G*_*B*_=(*B*,*T* *e* *r* *m*_*B*_,*E* *d* *g* *e*_*B*_), the similarity of these two graphs is measured through the weighted information contents of their common terms as well as their uncommon terms. We use Fig. 4 to illustrate this definition. The common terms between the two leaf term graphs *T* *G*_0048513_ and *T* *G*_0072358_ are shown in the dashed square box. The terms outside the square box are the uncommon terms of these two leaf term graphs. The MICA of these two term graphs is *G* *O*:0048731. The ancestry terms of the MICA are all in the square box, and all the descendant terms of MICA are outside the box.

The similarity of the two term graphs *T* *G*_*A*_ and *T* *G*_*B*_, denoted by *s* *i* *m*(*T* *G*_*A*_,*T* *G*_*B*_), is defined as

4 $$ {{}{\begin{aligned} {sim({TG}_{A}, {TG}_{B})\,=\,\frac{\sum\limits_{t\in common} \omega IC(t, G) }{\sum\limits_{t \in common} \omega IC(t, G) \,+\, \sum\limits_{t \in uncommon}\omega IC(t, G) }} \end{aligned}}}  $$

where, *common* is the set of common terms between *T* *G*_*A*_ and *T* *G*_*B*_, *uncommon* is the set of their uncommon terms, and *G* is the merged graph of *T* *G*_*A*_ and *T* *G*_*B*_.

The similarity *s* *i* *m*(*T* *G*_*A*_,*T* *G*_*B*_) ranges its values between 0 and 1. When the MICA of the two term graphs is the root node, the similarity between these two graphs is 0. If the two term graphs are the same, their similarity is 1.

As mentioned, the GO graph of a gene may contain multiple leaf nodes which correspond to multiple term graphs. Use *G*_1_ to denote the GO graph of gene *g*_1_ and *G*_2_ as the GO graph of gene *g*_2_. The similarity between *G*_1_ and *G*_2_ is measured by averaging the similarities of every leaf term graph of one GO graph (*G*_1_ or *G*_2_) with the other GO graph (*G*_2_ or *G*_1_). Assume *G*_1_ has *n*_1_ number of leaf terms ${LeafTerms}_{1}=\left \{l{\_{node}_{1}^{1}}, l{\_{node}_{1}^{2}}, \dots, l\_node^{n_{1}}_{1}\right \}$, and their leaf term graphs are denoted by $ TG({LeafTerms}_{1}) = \left \{{TG}_{\left (l{\_{node}_{1}^{1}}, G_{1}\right)}, {TG}_{\left (l{\_{node}_{1}^{2}}, G_{1}\right)},\dots, {TG}_{\left (l\_{node}_{1}^{n_{1}}, G_{1}\right)}\right \} $. Also assume *G*_2_ has *n*_2_ number of leaf terms ${LeafTerms}_{2}=\left \{l{\_{node}_{2}^{1}}, l{\_{node}_{2}^{2}}, \cdots, l\_node^{n_{2}}_{2}\right \}$, and their leaf term graphs are denoted by $TG({LeafTerms}_{2}) = \left \{{TG}_{\left (l{\_{node}_{2}^{1}}, G_{2}\right)}, {TG}_{\left (l{\_{node}_{2}^{2}}, G_{2}\right)}, \cdots, {TG}_{\left (l\_{node}_{2}^{n_{2}}, G_{2}\right)}\right \}$.

The similarity between *G*_1_ and *G*_2_, denoted by *s* *i* *m*(*G*_1_,*G*_2_), is given by

5 $$ {{}{\begin{aligned} sim(G_{1}, G_{2}) = \frac{\sum\limits_{tg \in TG\left({LeafTerms}_{1}\right)} sim(tg, G_{2}) \,+\, \sum\limits_{tg \in TG\left({LeafTerms}_{2}\right)} sim(tg, G_{1})}{n_{1} + n_{2}} \end{aligned}}}  $$

where, $sim(tg,G_{2})= \underset {1\leq i \leq n_{2}}{\max } sim(tg, {TG}_{(l{\_{node}_{2}^{i}}, G_{2})})$; and *s* *i* *m*(*t* *g*,*G*_1_) is similarly defined. We note that the maximal similarity of leaf-leaf term graph pairs is applied to measure the similarity between one leaf term graph and one GO graph.

### Clustering miRNAs for similar functions based on their target genes’ similarity/distance matrix

A miRNA usually has several target genes. In this work, the similarity between two miRNAs is measured by the similarity between the two sets of their target genes. We first introduce the similarity between a set of genes and a gene. Given a set of genes *G* *S*={*g*_1_,*g*_2_,…,*g*_*m*_} and a gene *g* ^′^, the similarity between *GS* and *g* ^′^ is given by

6 $$ sim(GS,g^{\prime})= \underset{1\leq i \leq m}{\max} sim(G(g_{i}), G(g^{\prime}))  $$

where *G*(*g*_*i*_) is the GO graph of *g*_*i*_, and *G*(*g* ^′^) is the GO graph of *g* ^′^.

An alternative method for measuring the similarity between a gene set and a gene is to take the average of the individual GO terms’ similarities. However, the average of the individual GO terms’ similarities can underestimate the true similarity between a gene set and a gene [15], as we use the similarity between a gene set and a gene to compute the similarity between two gene sets. This underestimated value will lower down the similarity between two gene sets.

Suppose we are given two miRNAs denoted by *R*_1_ and *R*_2_. Assume *R*_1_ has *s* number of target genes ${GS}_{1}=\{{g_{1}^{1}}, {g_{1}^{2}}, \dots, {g_{1}^{s}}\}$ and *R*_2_ has *k* number of target genes ${GS}_{2}=\{{g_{2}^{1}}, {g_{2}^{2}}, \dots, {g_{2}^{k}}\}$. The similarity of these two miRNAs *R*_1_ and *R*_2_ is defined as

7 $$ sim(R_{1},R_{2}) = \frac{\sum\limits_{1\leq i\leq k} sim({GS}_{1},{g_{2}^{i}}) + \sum\limits_{1\leq j\leq s}sim({GS}_{2},{g_{1}^{j}})}{s+k}  $$

The distance *dsim*, or dissimilarity, between two miRNAs *R*_1_ and *R*_2_ is computed by

8 $$ dsim(R_{1}, R_{2}) = 1- sim(R_{1}, R_{2})  $$

The dissimilarity between two miRNAs can be viewed as their distance, and thus it can be applied for clustering a group of miRNAs.

For a number *h* of miRNAs *R*_1_,*R*_2_,…,*R*_*h*_, a spectral clustering method [19] is applied to the dissimilarity matrix of these miRNAs to detect subsets of miRNAs which each have a similar function. The spectral clustering method is described as follows:

- For a set of data points *X*={*x*_1_,*x*_2_,…,*x*_*n*_}, construct a complete graph *SPG* in which the data point of *X* is the node of *SPG*. The weight $\omega _{(x_{i}, x_{j})}$ of each edge that connects with nodes *x*_*i*_ and *x*_*j*_, is defined as:
  9 $$ \omega_{(x_{i}, x_{j})} = e^{-\frac{\| x_{i}-x_{j}\|^{2}}{2\sigma^{2}}}  $$
  Let *Wam* denotes denote the weighted adjacency matrix of the graph *SPG*.
- Calculate the normalized Laplacian *L* from *Wam* and compute the first *k* eigenvectors of *L*. The *k* is the number of clusters. Then, the *k* eigenvectors can be used to construct a *n*∗*k* matrix *U*.
- The matrix *U* can be seen as a set of *n* data points under *k* features. Apply the *k*-means clustering algorithm to divide these data points.

For the *h* number of miRNAs *R*_1_,*R*_2_,…,*R*_*h*_, the weighted adjacency matrix *Wam* for the spectral clustering is determined by

10 $$ \omega_{(R_{i},R_{j})} = e^{-\frac{dsim(R_{i},R_{j})^{2}}{2\sigma^{2}}}  $$

where, 1≤*i*,*j*≤*h*.

The source code of spectral clustering is available at website http://sourceforge.net/projects/spectralcluster/?source=typ_redirect. Our source code of computing the weighted information contents can be downloaded from http://bioinformatics.gxu.edu.cn/bio/data/CWLan/spectralcode.tar.gz. Our results on clustering are available at http://bioinformatics.gxu.edu.cn/bio/data/CWLan/spectralresult.tar.gz.

There are two vital parameters in the spectral clustering method. The first one is *σ* in Eq. 10 and the other is the number of clusters. These two parameters have heavy influence to the clustering result. Traditional methods usually use several different choices of *σ* to test and choose the best *σ* by comparing the results. However, such approaches are time consuming. The selection of a good cluster number has been a challenging issue. In general, the cluster number relies on the user’s experience. In this paper, a self-tuning method is applied to decide an optimal value of *σ* and we also employ the eigenvalues of the normalized Laplacian matrix to determine an optimal number for the clusters.

**Self-tuning for the selection of** ***σ*** **.** Equation 10 uses the square of *σ*. The concern is that *σ* will be the same even though for computing two different data points. The self-tuning method employs two different *σ* values to calculate the weight of an edge. For the set of miRNAs *R*={*R*_1_,*R*_2_,…,*R*_*h*_}, the weight of its adjacency matrix by our self-tuning method is:

11 $$ \omega_{self}(R_{i},R_{j}) = e^{-\frac{dsim(R_{i},R_{j})^{2}}{2\sigma_{i}*\sigma_{j}}} i,j=1,2\ldots,h  $$

where *σ*_*i*_ is the average distance of *R*_*i*_ to all other miRNAs, given by

12 $$ \sigma_{i} = \frac{\sum_{j=1}^{h}dsim(R_{i},R_{j})}{h-1}  $$

**Fig. 5:**
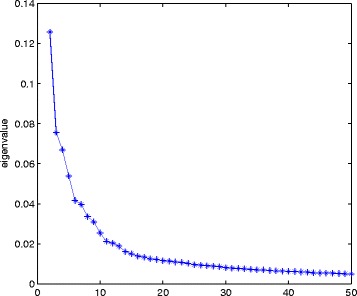
The first 50 largest eigenvalues

**Select the optimal cluster number.** An optimal number of clusters of the miRNAs is determined from the trend of the eigenvalues of the normalized Laplacian matrix *L*. Suppose these eigenvalues are *E* *i* *g*={*λ*_1_,*λ*_2_,…,*λ*_*h*_} sorted in a descending order. If the eigenvalue *λ*_*k*+1_ (1≤*k*<*h*) is very small and the trend of the subsequent eigenvalues goes stable, then the number of clusters can be set as *k*. If the differences between two consecutive eigenvalues are very small, we said that the trend of the consecutive eigenvalues goes stable. Figure 5 presents the first 50 largest eigenvalues of the normalized Laplacian matrix *L* of the miRNA data set used in the second section of “Data Sets and Definitions Related to GO Graphs". Therefore, the cluster number 13 is selected.

## Results

Our method was applied to the data set of 289 Human miRNAs downloaded from http://mirtarbase.mbc.nctu.edu.tw/ to cluster their function groups. (The details of the data set have been described in the second Section.) 265 of these miRNAs are associated with a disease. The disease information of the remaining 24 miRNAs are not available from the database at the time of this work. Instead their functions were predicted by our clustering method. We report four parts of computational results in this section. The first part shows the importance of our edge weights to the information contents of term nodes in GO graphs. The second part selects a good edge relationship weight and discusses the effect of the edge relationship weight on the cluster number. The third part compares our method with three existing methods to understand our superior clustering performance. The forth part reports the function annotation results for new miRNAs by our clustering method.

### The effect of edge weights on the information contents of term nodes

**Fig. 6:**
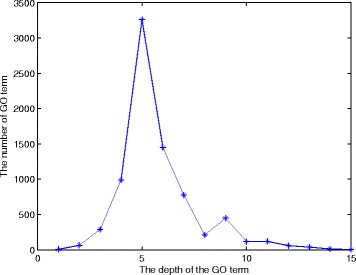
The numbers of leaf terms at different levels of the GO trees

The results in this section explain why we introduce the edge weight of a term to adjust the information content of the term (using our Eqn. 1). Figure 6 presents the numbers of leaf terms of the GO trees when the term level varies. The majority of these leaf terms are at level 5. By the traditional definition of information content, all these leaf terms have the same *IC* value, although they are at different levels of the trees. This is why we use an edge weight to adjust the information content of a leaf term and make it proportional to the distance of the path from the leaf node to the MICA. Namely, a leaf term having a far distance to the MICA should contain more information than a leaf term closer to the MICA.

**Fig. 7:**
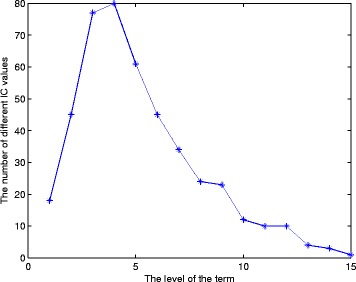
The numbers of different *IC* values at the same levels of the GO trees

Figure 7 shows the numbers of different *IC* values for the terms at the same levels of the GO trees, where the *IC* values are computed according to our definition of information content. For example at level 4 of these GO trees, there are many terms having different *IC* values. These terms should have different *IC* values, as they contain different numbers of descendants. The traditional edge-based method [18] assigns the same weights to the terms at the same level. The combination of *IC* value and the edge weight by our Eqn. 3 overcomes this weak point of the node-base method [12] and the edge-based method [18].

### Effect of edge relationship weights on the number of clusters

We have proposed to use edge relationships in GO trees to define an edge weight. As this work focuses on the prediction and annotation of miRNA functions, we use the molecular functions of GO terms which only have relationship *is_a* between GO terms. We tested and compared the effectiveness of 9 different weights of the *is_a* relationship from 0.1 to 0.9 with step increase of 0.1 on the function prediction performance for the 265 miRNAs.

An accuracy rate is used to measure the quality of the clustering results. It is defined as the proportion of miRNAs in a cluster which are associated with the same disease:

13 $$ accr(disease, C) = \frac{nm(disease)}{\| C\|}  $$

where *n* *m*(*d* *i* *s* *e* *a* *s* *e*) is the number of miRNAs associated with the *disease*, and ∥*C*∥ is the total number of miRNA in the cluster *C*. Usually, a cluster of miRNAs formed by computational methods can have diverse proportions of miRNAs each sharing a different disease. We used the accuracy of the prevailing disease to represent the accuracy rate of the cluster. A high accuracy of a cluster means that many miRNAs associated with the same disease are clustered into the same group, implying the weight of the *is_a* relationship is properly assigned for the function prediction of new miRNAs.

The breast cancer, stomach cancer, and hepatocellular carcinoma were three diseases which are most prevailing in three clusters for all of the situations of the relationship weight from 0.1 to 0.9. The detailed accuracy rates are presented in Table 2. We found that 0.8 was a good relationship weight.

**Table 2:** Accuracy rates of three different clusters by setting 9 different edge relationship weights

Relationship Weight group	0.1 group	0.2 group	0.3 group	0.4 group	0.5 group			
Breast Neoplasms Cluster	35/43=0.814	35/42=0.833	35/42=0.833	37/44=0.841	30/35=0.857			
Hepatocellular Carcinoma Cluster	14/25=0.56	10/19=0.526	16/27=0.593	14/24=0.583	12/23=0.522			
Stomach Neoplasms Cluster	15/27=0.55	13/24=0.542	13/26=0.500	15/25=0.600	14/26=0.538			
Relationship Weight group	0.6 group	0.7 group	0.8 group	0.9 group				
Breast Neoplasms Cluster	33/40=0.825	36/43=0.837	36/43=0.837	36/43=0.837				
Hepatocellular Carcinoma Cluster	12/27=0.444	17/23=0.739	18/24=0.750	11/26=0.423				
Stomach Neoplasms Cluster	11/24=0.458	17/28=0.607	17/27=0.630	14/26=0.538				

**Fig. 8:**
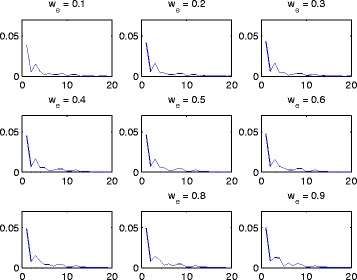
The consecutive differences of the first 20 largest eigenvalues (i.e., the differences between the *k*th and (*k*+1)th eigenvalue) under the setting of different edge relationship weights

Figure 8 shows that the eigenvalues from the 10th to the 20th become very stable (i.e., the difference between two consecutive eigenvalues becomes close to 0) under all situations of the relationship weight from 0.1 to 0.9. As discussed above, cluster number 13 was chosen to group miRNAs of similar functions. It can be seen that the effect of the edge relationship weights on the cluster number is very small.

### Clustering performance comparison with existing methods

We compared our method with three literature methods to understand the grouping performance for miRNAs of similar functions. The three literature methods are a node-based approach by Lin [12], edge-based approach by Viktor [20], and hybrid approach by Wang [21].

The performance by each clustering method is reported in Table 3.

**Table 3:** Accuracy rates on the three clusters by different methods

Method	Our method	Lin’s method	Viktor’s method	Wang’s method				
Breast Neoplasms Cluster	36/43=0.837	24/29=0.829	26/31=0.839	36/43=0.837				
Hepatocellular Carcinoma Cluster	18/24=0.750	20/24=0.833	15/22=0.682	13/21=0.619				
Stomach Neoplasms Cluster	17/27=0.630	12/23=0.522	15/29=0.517	14/30=0.467				

For the Breast Neoplasms Cluster, all the four methods have very close and competitive accuracy. For the Hepatocellular Carcinoma Cluster, Lin’s method has the largest number of miRNAs and the highest accuracy. Our method has the second largest number of miRNAs on the hepatocellular carcinoma cluster and the second highest accuracy. For the Stomach Neoplasms cluster, our method yields the largest number of miRNAs and the highest accuracy rate. Overall, our method generates the best accuracy for the union of the three clusters, and has the largest coverage of the miRNAs (the total number of miRNAs in the clusters). Wang’s method has the same coverage of 94 miRNAs as ours, but its accuracy is about 30% lower. Lin’s method has a similar overall accuracy as ours, but its coverage is about 20% smaller.

### Co-regulating miRNAs and function annotations for new miRNAs

As suggested, miRNAs clustered into the same group should have similar functions. Some of our experiments have verified this point. For example, the pair of miRNA-519d and miRNA-216a in the Hepatocellular Carcinoma cluster have a similar function. In fact miRNA-519d [22] and miRNA-216a [23] had been both found to up-regulate PTEN in hepatocellular carcinoma cells. Another example is from the Breast Cancer cluster about the pair of miRNA-205 and miRNA-145. miRNA-205 is involved in the regulation of breast cancer [24], while miRNA-145 also plays a vital role in regulating breast cancer [25]. In the Stomach Cancer cluster, it can be confirmed that miRNA-150 is related to stomach caner [26] and miRNA-106a is also related to this cancer [27]. Many previous studies have indicated that multiple miRNAs can work together to effect cancer formation [28]. Our method to identify these miRNA clusters can assist in investigating this mechanism [29].

The functions/disease information of some miRNAs (24) of our 289-miRNA data set are still un-annotated in the HMDD database. However these un-annotated miRNAs can be clustered into some groups by our method, and their functions can be annotated according to the prevailing functions of the groups:

- 5 of the 24 un-annotated miRNAs are grouped into the breast cancer cluster (miRNA-129, miRNA-135a, miRNA-196a, miRNA-5787, and miRNA-9),
- 4 are grouped in the stomach cancer cluster (miRNA-103a, miRNA-181a, miRNA-19b, and miRNA-519a),
- 2 are in the Hepatocellular Carcinoma cluster (miRNA-515 and miRNA-639),
- 8 are classified into the Ovarian cancer cluster (miRNA-512, miRNA-518a, miRNA-521, miRNA-644a, miRNA-876, miRNA-886, miRNA-892b, miRNA-153),
- 2 are clustered in the Prostatic cancer cluster (let-7f and miRNA-219a), and
- 3 are in the Colorectal cancer cluster (miRNA-30c, miRNA-181b, and miRNA-513a).

We have found evidence to support our annotation for some of these miRNAs, for example, miRNA-9 which is asigned into the breast cancer cluster. In fact, recent research shows that miRNA-9 is a potential biomarker for breast cancer [30]. The miRNA-129 is also predicted as a regulator in breast cancer by our method. A recent study can support this prediction: miRNA-129 is down-regulated in breast cancer and has effect on breast cancer migration and motility [31]. It has also been claimed that miRNA-135a is very critical in regulating breast cancer — miRNA-135a can bind to gene ESRR1 which is related with the breast cancer [32].

For the un-annotated miRNAs in the Stomach cancer cluster, it has been found that miRNA-181a is up-regulated in stomach cancer and has effects on cell proliferation in stomach cancer [33]. Literature work also supports that miRNA-19b and miRNA-519a are associated with stomach cancer [34, 35]

In the ovarian cancer cluster, two studies have shown that miRNA-521 and miRNA-153 are indeed associated with the ovarian cancer [36, 37]. In the Colorectal Cancer cluster, three un-annotated miRNAs miRNA-30c, miRNA-181b, and miRNA-513a can be verified that they are related with this cancer [38–40].

## Discussion and conclusion

A variety of methods have been developed to study the functional roles of miRNAs by dividing them into functional groups. For example, Kaczkowski applies the miRNAs’ sequence and their secondary structure to cluster miRNAs [41]. However, the miRNAs with a high similarity in sequence/structure cannot guarantee similar functions. Thus, the target genes of miRNAs have been taken as an alternative information source to investigate miRNAs functions.

One of the most prevalent comparative methods for the similarity of target genes is GO graph. The approaches can be classified into two categories: (1) those node-based methods and edge-based methods using GO terms, and (2) pairwise methods and groupwise methods using gene products. Typical node-based methods include Resnik’s [42], Lin’s [12], and Jiang and Conrath’s algorithm [43]. This kind of method applies the *IC* for measuring the similarity of two GO graphs.

The Resnik’s method uses only the MICA to measure the similarity between two terms. However, this kind of method neglects the dissimilarity of two terms. Other node-based methods consider both the *IC* value of terms as well as the MICA of two GO graphs, such as Lin’s method and Jiang and Conrath’s method. Although node-based methods are useful in measuring similarity of terms, the original *IC* value relies on a specific corpus and the structure of the GO graph is largely ignored.

The edge-based methods utilize the length between root nodes and terms. The edge-based method applies the length between root node to the MICA and the distances between the MICA and the leaf terms. The edge method reflects the structure of the GO graph. It assumes all edges have equal weight. However, edges in GO graphs can describe two different relationships (is_a and part_of), which should be assigned with different weights. In addition, the edge-based methods view the weight of all GO terms as the same. However, it is reasonable that a term should have lower weight if it is closer to the root node of the GO graph.

Both edge-based methods and node-based methods have their own advantages. Thus, some methods combine the weight of the term and the distance between two terms to measure the similarity of two GO graphs. This kind of method is called hybrid methods. For example, Sevilla applies the *edge* and the *IC* to measure the similarity of two nodes [44]. While this kind of the method always ignores the relationship of the edge. Wang’s method [21] is a typical hybrid method that takes the relationship of the edge into consideration. However, if two term pairs have the same structure, they will have the same similarity value.

This work has introduced a new GO-based method to cluster miRNAs for similar functions. A weighted information content is proposed to measure the importance of a term in a GO graph. Its key idea is to integrate the descendant-based information content, the depth of the term, and the relationships of the edges in the path from the term to the root node. Our weighted information content can overcome some limitations of the conventional node-based and edge-based approaches. The similarity between two GO graphs is based on the weighted information contents of the common terms relative to the information contents of the uncommon terms. These similarities are transferred to estimate the similarities of miRNAs. A spectral clustering method has been applied to the similarity/distance matrix of a set of 289 miRNAs for function grouping. Compared with three state-of-the-art clustering methods, our method show better performance in accuracy to measure the similarity/distance between miRNAs. Our method is also useful for the discovery of co-regulating miRNAs and the function annotation of new miRNAs.
